# The transmission dynamics and diversity of human metapneumovirus in Peru

**DOI:** 10.1111/irv.12537

**Published:** 2018-03-30

**Authors:** Simon Pollett, Nidia S. Trovão, Yi Tan, John‐Sebastian Eden, Rebecca A. Halpin, Jayati Bera, Suman R. Das, David Wentworth, Victor Ocaña, Silvia M. Mendocilla, Carlos Álvarez, Maria E. Calisto, Josefina Garcia, Eric Halsey, Julia S. Ampuero, Martha I. Nelson, Mariana Leguia

**Affiliations:** ^1^ University of Sydney Sydney NSW Australia; ^2^ Walter Reed Army Institute of Research Silver Spring MD USA; ^3^ National Institutes of Health Bethesda MD USA; ^4^ Mount Sinai University New York NY USA; ^5^ J Craig Venter Institute Rockville MD USA; ^6^ Pachitea Health Center Ministerio de Salud Piura Peru; ^7^ Hospital Daniel Alcides Carrión Ministerio de Salud Callao Peru; ^8^ Dirección Regional de Salud Loreto Loreto Peru; ^9^ Hospital Nacional Edgardo Rebagliati Martins Seguro Social de Salud‐EsSalud Lima Peru; ^10^ US Naval Medical Research Unit No‐6 Lima Peru; ^11^ Present address: Vanderbilt University Medical Center Nashville TN USA

**Keywords:** human metapneumovirus, Peru, phylogeography, transmission dynamics

## Abstract

**Background:**

The transmission dynamics of human metapneumovirus (HMPV) in tropical countries remain unclear. Further understanding of the genetic diversity of the virus could aid in HMPV vaccine design and improve our understanding of respiratory virus transmission dynamics in low‐ and middle‐income countries.

**Materials & Methods:**

We examined the evolution of HMPV in Peru through phylogenetic analysis of 61 full genome HMPV sequences collected in three ecologically diverse regions of Peru (Lima, Piura, and Iquitos) during 2008‐2012, comprising the largest data set of HMPV whole genomes sequenced from any tropical country to date.

**Results:**

We revealed extensive genetic diversity generated by frequent viral introductions, with little evidence of local persistence. While considerable viral traffic between non‐Peruvian countries and Peru was observed, HMPV epidemics in Peruvian locales were more frequently epidemiologically linked with other sites within Peru. We showed that Iquitos experienced greater HMPV traffic than the similar sized city of Piura by both Bayesian and maximum likelihood methods.

**Conclusions:**

There is extensive HMPV genetic diversity even within smaller and relatively less connected cities of Peru and this virus is spatially fluid. Greater diversity of HMPV in Iquitos compared to Piura may relate to higher volumes of human movement, including air traffic to this location.

## 1 INTRODUCTION

Human metapneumovirus (HMPV) causes substantial morbidity particularly in the extremes of age and in the immunocompromised.^1^ Despite the burden of disease due to this pathogen, there is a limited knowledge of the diversity and transmission dynamics of this virus in Peru and other tropical countries due to the low availability of molecular data.^2^ Further understanding of the genetic diversity of the virus could aid in HMPV vaccine design and improve our understanding of respiratory virus transmission dynamics in low‐ and middle‐income countries (LMIC). We therefore examined the evolution of HMPV in Peru through phylogenetic analysis of 61 full‐genome HMPV sequences collected in three ecologically diverse regions of Peru (Lima, Piura, and Iquitos) during 2008‐2012, comprising the largest dataset of HMPV whole genomes sequenced from any tropical country to date (Table 1). Using these data, we assessed (i) the extent of genetic diversity in each location, (ii) the extent of gene flow of HMPV between Peru and other regions globally, and (iii) whether viruses from three ecologically diverse Peruvian cities with distinctly different seasonal patterns of influenza‐like illnesses were more similar to each other than from the global gene pool.

**Table 1:** Total number of HMPV sequences analyzed in Peru and flight patterns

	Location		
	Lima	Piura	Iquitos
Number of sequences analyzed by year			
2008	0	5	2
2009	1	13	3
2010	2	4	10
2011	0	8	8
2012	1	1	3
All years	4	31	26

	Lima	Iquitos	Piura
Annual number of air passengers arriving into Peruvian locale^a^			
Peru origin	4 714 351	510 651	413 850
Global origin	4 089 712	4422	0
Total (any origin)	8 804 063	515 073	413 850

HMPV, human metapneumovirus.

a 2015 data, derived from http://www.corpac.gob.pe.

## 2 METHODS

### 2.1 Study population, fieldwork, and initial laboratory procedures

Study respiratory specimens were derived from influenza‐like illness (ILI) sentinel and active surveillance studies in three Peruvian cities between 2008 and 2012 which yielded 87 HMPV PCR‐positive specimens. Lima (n = 15 specimens) is the temperate urban capital and major air‐hub of Peru, with a large population (~8.5 million). Lima receives >4 million international flights annually, and is a hub of transportation within Peru (Table 1) (3). Piura (n = 34 specimens) is a semi‐arid regional capital of northern Peru with a fraction of Lima's population (377 000) and greatly reduced volume of air traffic (Table 1).^3^ Iquitos (n = 38 specimens) is similar to Piura in terms of demography (population = 370 000), but is located deep in the Amazonian tropics and accessible only by air or river.^3^ Previous studies have demonstrated that ILI activity peaks in winter in Lima, but is less seasonal in Piura and Iquitos, which tend to have year‐round incidence (Y.Tinoco unpublished data). The field and laboratory methods of the surveillance studies yielding this biobank of HMPV‐positive specimens from these three cities are described in full detail elsewhere.^2^, ^4^, ^5^

Briefly, those presenting to healthcare settings in Lima (Hospital Nacional Daniel Alcides Carrion and Hospital Edgardo Rebagliati), Piura (Pachitea Health Center), and Iquitos (Hospital Regional, Hospital Apoyo, Quistococha Health Center, Tupac Amaru Health Center, San Antonio Health Center, Moronacocha Health Center and Bellavista‐Nanay Health Center) were screened for study enrollment and included if they met criteria for influenza‐like‐illness, defined as fever 38°C plus either cough or sore throat. Naso‐oropharyngeal swabs were selected from eligible participants, in addition to data on age, sex, and whether the patient was managed as an inpatient or outpatient at the time of collection. Importantly, there was heterogeneity in specimen collection by location with a highly engaged collaborator who provided a relatively large number of respiratory samples for Piura.

In addition to sentinel surveillance in each of the three Peruvian locations, specimens were also leveraged from active influenza‐like illness surveillance in Iquitos as described by Forhsey et al.^5^ Briefly, enrolled community‐based participants were visited at their residence 3 times per week by healthcare workers who elicited whether participants had ILI as per the definition used for sentinel surveillance, with naso‐oropharyngeal swabs and demographic data collected from eligible participants.

These specimens were sent in viral transport media at −70°C transport media to US Naval Medical Research Unit No. 6 (NAMRU‐6) in Callao, Peru, where they underwent initial screening for a broad number of respiratory viruses using Madin‐Darby canine kidney (MDCK), African green monkey kidney (Vero76 and VeroE6), and Rhesus monkey kidney (LLCMK2) cell lines. Upon demonstration of cytopathic effect, respiratory virus identification was performed by direct immunofluorescence (D3 DFA Respiratory Virus Diagnostic Hybrids; Athens, OH), for adenoviruses, influenza A virus, influenza B virus, parainfluenza viruses, respiratory syncytial respiratory viruses, and HMPV.^2^ Those specimens positive for HMPV by immunofluorescence then underwent confirmatory testing by PCR as described by Garcia et al.^2^ Briefly, nucleic acid extraction was performed by QIAamp viral RNA kit (Qiagen; Valencia, CA) from the naso‐oropharyngeal swab solution before reverse transcriptase‐PCR using a SuperScript III One‐Step RT‐PCR System (Invitrogen; San Diego, CA) in a thermocycler 7700 (Applied Biosystems; Foster City, CA). The primers used were specific to the G gene segment: hMPVG1F (ATG GAG GTG AAA GTG GAG AAC AT) and hMPVG1R (GTG GAT TCA TTG AGA GGA TCC AT). For further verification, the isolates underwent also a PCR with N‐gene–specific primers hmpv1 (CCC TTT GTT TCA GGC CAA) and hmpv2 (GCA GCT TCA ACA GTA GCT). Original naso‐oropharyngeal swab specimens which were positive for HMPV were then biobanked.

### 2.2 Whole‐genome sequencing

Banked original naso‐oropharyngeal swab respiratory specimens which were known HMPV‐positive by PCR by the above methods were sent (at −80°C) for RNA extraction and sequencing of the full HMPV whole genome at J. Craig Venter Institute. Extraction of the viral RNA was performed using 140 μL of the respiratory sample using the ZR 96 Viral RNA kit (Zymo Research Corporation, Irvine, CA, USA). Four forward reverse transcription (RT) (MPV_6F, ACGCGAAAAAAAC; MPV_3717F, ATAACACCAGCAATAT; MPV_7773F; GAAGTAATAAGAACYGG; and MPV_9696F, AAGGTRATTCAATCTG) MPV_7773F; GAAGTAATAAGAACYGG; and MPV_9696F, AAGGTRATTCAATCTG) and four sets of PCR primers were manually picked (see Table S3) from primers designed across a consensus of complete HMPV genome sequences using JCVI's automated primer design tool.^6^ The four forward RT primers were diluted to 2 μmol/L and pooled in equal volumes. cDNA was generated from 4 μL undiluted RNA, using the pooled forward primers and SuperScript III Reverse Transcriptase (Thermo Fisher Scientific, Waltham, MA, USA). Four‐independent PCR reactions were performed on 2 μL of cDNA template using either AccuPrime Taq DNA Polymerase (Thermo Fisher Scientific) or Phusion High Fidelity DNA Polymerase (New England Biolabs, Ipswich, MA, USA) to generate four overlapping ~4‐kb amplicons across the genome. Amplicons were verified on 1% agarose gels, and excess primers and dNTPs were removed by treatment with Exonuclease I (New England Biolabs) and shrimp alkaline phosphatase (Affymetrix, Santa Clara, CA, USA) for 37°C for 60 minutes, followed by incubation at 72°C for 15 minutes. Amplicons were quantitated using a SYBR Green dsDNA detection assay (SYBR Green I Nucleic Acid Gel Stain, Thermo Fisher Scientific), and all four amplicons per genome were pooled in equal concentration..

For samples sequenced using the Ion Torrent PGM (Thermo Fisher Scientific), 100 ng of pooled DNA amplicons was sheared for 7 minutes, and Ion Torrent‐compatible barcoded adapters were ligated to the sheared DNA using the Ion Xpress Plus Fragment Library Kit (Thermo Fisher Scientific) to create 400‐bp libraries. Libraries were pooled in equal volumes and cleaned with Ampure XP reagent (Beckman Coulter, Inc., Brea, CA, USA). Quantitative PCR was performed on the pooled, barcoded libraries to assess the quality of the pool and to determine the template dilution factor for emulsion PCR. The pool was diluted appropriately and amplified on Ion Sphere Particles (ISPs) during emulsion PCR on the Ion One Touch 2 instrument (Thermo Fisher Scientific). The emulsion was broken, and the pool was cleaned and enriched for template‐positive ISPs on the Ion One Touch ES instrument (Thermo Fisher Scientific). Sequencing was performed on the Ion Torrent PGM using 316v2 or 318v2 chips (Thermo Fisher Scientific).

For samples requiring extra coverage, in addition to Ion Torrent sequencing, Illumina libraries were prepared using the Nextera DNA Sample Preparation Kit (Illumina, Inc., San Diego, CA, USA) with half reaction volumes. Briefly, 25 ng of pooled DNA amplicons was tagmented at 55°C for 5 minutes. Tagmented DNA was cleaned with the ZR‐96 DNA Clean & Concentrator Kit (Zymo Research Corporation) and eluted in 25 μL resuspension buffer. Illumina sequencing adapters and barcodes were added to tagmented DNA via PCR amplification, where 20 μL tagmented DNA was combined with 7.5 μL Nextera PCR Master Mix, 2.5 μL Nextera PCR Primer Cocktail, and 2.5 μL of each index primer (Integrated DNA Technologies, Coralville, IA, USA) for a total volume of 35 μL per reaction. Thermocycling was performed with 5 cycles of PCR, as per the Nextera DNA Sample Preparation Kit protocol (3 minutes at 72°C, denaturation for 10 seconds at 98°C, annealing for 30 seconds at 63°C, and extension for 3 minutes at 72°C) to create a dual‐indexed library for each sample. After PCR amplification, 10 μL of each library was pooled into a 1.5‐mL tube, and the pool was cleaned two times with Ampure XP reagent (Beckman Coulter, Inc.) to remove all leftover primers and small DNA fragments. The first cleaning used a 1.2 × volume of the Ampure reagent, while the second cleaning used a 0.6 × volume of the Ampure reagent. The cleaned pool was sequenced on the Illumina MiSeq v2 instrument (Illumina, Inc.) with 300‐bp paired‐end reads.

Sequence reads were sorted by barcode, trimmed, and de novo assembled using CLC Bio's clc assembler program, formerly known as clc novo assembly (http://resources.qiagenbioinformatics.com/manuals/clcgenomicsworkbench/852/index.php?manual=De_novo_assembly.html), and the resulting contigs were searched against custom, full‐length HMPV nucleotide databases to find the closest reference sequence. All sequence reads were then mapped to the selected reference HMPV sequence using CLC Bio's clc_mapper_legacy, formerly called as clc_ref_assemble_long program (http://resources.qiagenbioinformatics.com/manuals/clcassemblycell/current/index.php?manual=Options_clc_mapper_legacy.html). At loci where both Ion Torrent and Illumina sequence data agreed on a variation (compared with the reference sequence), the reference sequence was updated to reflect the difference. A final mapping of all next‐generation sequences to the updated reference sequences was performed with CLC Bio's clc_mapper_legacy program. Curated assemblies were validated and annotated with the viral annotation software called Viral Genome ORF Reader, VIGOR 3.0 35, before submission to GenBank. VIGOR was used to predict genes, perform alignments, ensure the fidelity of open reading frames, correlate nucleotide polymorphisms with amino acid changes, and detect any potential sequencing errors. The annotation was subjected to manual inspection and quality control before submission to GenBank. All sequences generated as part of this study were submitted to GenBank as part of the Bioproject ID PRJNA237298.

### 2.3 Evolutionary analyses

To understand how HMPV in Peru relates to viral populations sampled globally, a global background dataset of HMPV F‐gene sequences was downloaded from GenBank, including 307 viruses collected between 1998 and 2013 with a minimum length of 1100 nt (Table S1). These sequences were aligned with the 61 Peruvian full F‐gene sequences using MUSCLE^7^ and manually edited using MEGA 6.0^8^ to yield a final dataset of 368 sequences. From this global alignment, the best‐fit nucleotide substitution model was determined to be GTR + Γ with a proportion of invariant sites based on the Akaike information criterion available in JModelTest 2.^9^ A phylogeny was inferred using the maximum‐likelihood (ML) methods available in RAxML v7.2.6.^10^ Statistical robustness was assessed by bootstrap resampling (500 replicates).

Studying the spatial dynamics of HMPV in Peru is complicated by the low number of samples available from Lima, the largest and most interconnected city in the country (Table 1). Therefore, we examined only broad spatial patterns in our phylogeographic analysis, in which we reconstructed the spatial dynamics of HMPV using a discrete phylogeographic analysis, parametrized with a non‐reversible continuous‐time Markov chain (CTMC) process and Bayesian stochastic search variable selection (BSSVS) integrated in the Bayesian Evolutionary Analysis Sampling Trees (BEAST) package. These analyses were performed only for the A2 clade for both the whole‐genome dataset and the F‐gene dataset, due to deep evolutionary divergence between HMPV subtypes and as the majority of Peruvian sequences belonged to this clade (whole‐genome dataset: Peruvian sequences = 51, non‐Peruvian sequences = 20; F‐gene dataset: Peruvian sequences = 53, non‐Peruvian sequences = 118).

### 2.4 Ethical considerations

The Naval Medical Research Unit Number‐6 Institutional Review Board approved the field studies that yielded the HMPV‐positive respiratory specimens.

## 3 RESULTS

Of the 87 HMPV‐positive specimens, 61 were able to be successfully sequenced (Lima n = 4 of 15 specimens, Iquitos n = 26 of 38 specimens, Piura = 31 of 34 specimens). The median age of the 61 cases from which HMPV sequence data were derived was 4 years (IQR = 1‐6, range = <1 year old to 68 years old) and 49% (30/61) were male. Five of the cases were admitted as an inpatient at the time of specimen collection, a further 42 cases presented as an outpatient during enrollment, and a final 14 were detected in the community during active surveillance.

**Figure 1:**
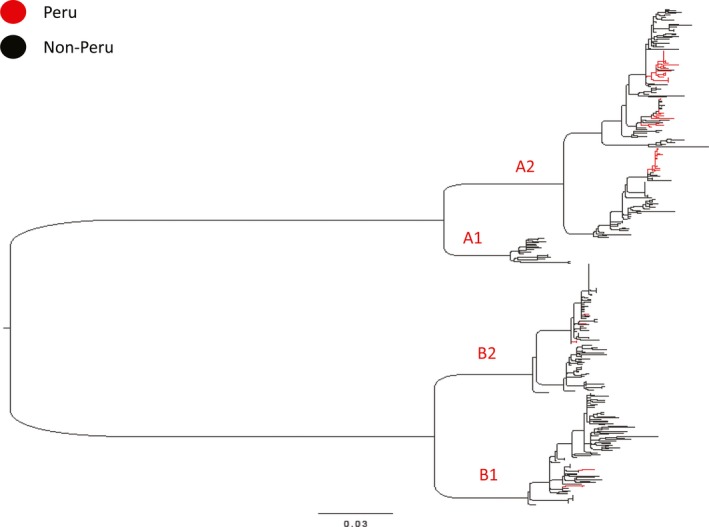
Maximum likelihood inferred phylogeny of Peru (red) and reference global HMPV F‐gene sequences (black), rooted by avian pneumovirus group C outgroup (GenBank accession AY579780, removed for clarity). Scale bar indicates number of nucleotide substitutions per site, and major clade identities are indicated in red (A1, A2, B1, B2). All taxa labels and bootstrap values are indicated in Figure S4

Of the four major HMPV lineages defined globally (A1, A2, B1, and B2 subclades), all but A1 viruses were identified in Peru (Figure 1, Table 2, Figures S1, S2, and S3 present trees for these A2, B1, and B2 HMPV clades, respectively. Figure S4 displays the global phylogeny with all labeled taxa and bootstrap annotations). The A2, B1, and B2 subclades were observed even in the more isolated locales of Iquitos and Piura, and several clusters with high bootstrap support were identified that included viruses from all 3 Peruvian cities, an indication of intracountry movement of viruses between locations with markedly different climates.

**Table 2:** HMPV subtype distribution and gene flow into Peruvian locations

	Lima	Iquitos	Piura
HMPV subgroups present by year and location			
2008	‐	A2, B1	A2, B1
2009	A2	A2, B1	A2, B2
2010	A2	A2, B2	A2, B2
2011	‐	A2	A2
2012	A2	A2	A2
All years	A2	A2, B1, B2	A2, B1, B2
Putative new HMPV introductions by year and location and subgroup^a^			
2008	‐	2 (A2[1],B1[1])	2 (A2[1],B1[1])
2009	1 (A2[1])	2 (A2[1],B1[1])	2 (A2[1],B2[1])
2010	1 (A2[1])	3 (A2[2],B2[1])	3 (A2[2],B2[1])
2011	‐	1 (A2[1])	0
2012	1 (A2[1])	1 (A2[1])	0
All years	3	9	7

Origin state	Location of importation		
	Lima	Iquitos	Piura
Whole‐genome “Markov jump” counts into Peruvian cities (A2 lineage)^b^			
Peru	4.02	9.42	0.26
Global	0	0.07	3.02
Any	4.02	9.49	3.28
F‐gene “Markov jump” counts into Peruvian cities (A2 lineage)^b^			
Peru	4.06	7.3	1.5
Global	0.01	0.71	2.79
Any	4.07	8.01	4.29

HMPV, human metapneumovirus.

a Derived from maximum‐likelihood phylogeny (Figure 1). For each year and location, the distribution of new introductions by subgroup is indicated in brackets.

b Performed on data from the A2 lineage only.

We found that HMPV is frequently introduced into each of the 3 Peruvian localities, including undersampled Lima, based on the estimated number of location transitions (“Markov jumps”) on the trees that were estimated using stochastic mapping techniques (Table 2). Estimates of viral gene flow into a location were not related to the number of sequences available from that location. In fact, the lowest viral gene flow was observed in Piura (Table 2), for which the largest number of sequences was available (Table 1). The phylogeographic analysis also suggested that, overall, HMPV epidemics in Peru were more likely to be seeded from other locations in Peru rather than from a global gene pool (Table 2). These findings were consistent with the phylogeographic results on the F‐gene dataset (Table 2), as well as the number of introductions estimated from the ML tree (Table 2). A higher degree of geographic clustering also was observed Piura compared to Iquitos, as assessed using the phylogeny‐trait association test (Bayesian Tip‐association Significance [BaTS])^11^ (*P *<* *.01, Table S2), which was conducted upon a posterior distribution of trees generated in BEAST, as described above. The evolutionary rate of the F‐gene estimated under a strict clock (9.70 × 10^−4^ ‐ 95% highest posterior density (HPD): 5.37 × 10^−4^‐1.4 × 10^−3^) was similar to prior estimates (7.1‐9 × 10^−4^) and to estimates using a relaxed clock (1.49 × 10^−3^‐95% HPD: 5.46 10^−4^‐3.05 × 10^−3^).^1^, ^12^

## 4 CONCLUSIONS

By sequencing 61 HMPV specimens in Peru, we have generated the largest dataset of whole‐genome sequences from any tropical country for this important respiratory pathogen. Importantly, the case characteristics yielding these data (overall skew toward pediatric age‐groups who are frequently managed on an ambulatory basis rather than requiring inpatient admission) are similar to those noted in HMPV study in other regions^1^ and were derived from both populations presenting to health care as well as those actively identified in the community via active surveillance, thereby improving the generalizability of our results. Notably, the genetic diversity of HMPV in Peru spans almost the entire known global HMPV diversity, due to widespread gene flow within Peru and between Peru and other regions. The lack of data available from Lima has limited our ability to infer viral movements within Peru at a refined scale, and we suspect that increased sequencing in Lima will be central to understanding HMPV dynamics in Peru, given Lima's volume of domestic and international air traffic. This geospatially skewed data due to ascertainment bias (heterogeneity in specimen collection which doesn't correlate with disease incidence) are an unfortunately common limitation of many spatial phylodynamic studies of other respiratory RNA viruses.^13^ We tried to explicitly account for this spatial skew and ascertainment bias in our analysis. Specifically, we did not make any specific conclusions of the extent of viral diffusion from Lima to Piura or Lima to Iquitos. Nor did we estimate the absolute viral traffic in Lima or compare it to Piura and Iquitos.

Still, our conservative approach allowed us to identify extensive genetic diversity even within the smaller and relatively less connected cities. We further showed that there is limited persistence of HMPV in tropical locations and that this virus is spatially fluid. Furthermore, we demonstrate that Iquitos experiences greater HMPV traffic than the similarly sized city of Piura, which may relate to greater volumes of air traffic through Iquitos (Table 1), and indicates there is some correlation between human movement and HMPV dispersal, at least in these two locations that were well‐sampled. The role of human movement in putatively driving greater than expected viral traffic through a locale has also been noted in recent phylogeographic studies of H3N2 influenza A virus in Peru.^14^

The general lack of sustained persistence of HMPV in any of the three locations across years indicates that that viral diversity in any locale in Peru is likely maintained by continual migration and re‐introduction (from either within or outside of Peru). This has also been demonstrated for both influenza and respiratory syncytial virus (RSV) epidemics in Peru.^14^, ^15^ While epidemics in discrete Peruvian locales were shown to be more likely to be seeded from elsewhere in Peru than a non‐Peruvian country, a caveat is that countries immediately bordering Peru were undersampled and such conclusions about the spatial epidemiology of HMPV in tropical regions should be carefully reproduced in other settings as global HMPV genome sequence databases increase.

## DISCLAIMER

The views expressed in this article are those of the authors and do not necessarily reflect the official policy or position of the Department of the Navy, Department of Defense, nor the U.S. Government. Several of the authors are US Government Employees. This work was prepared as part of their official duties. Title 17 U.S.C. § 105 provides that “Copyright protection under this title is not available for any work of the United States Government.” Title 17 U.S.C. §101 defines a U.S. Government work as a work prepared by a military service member or employee of the U.S. Government as part of that person's official duties. The authors declare no conflict of interests.
