# Supplementary figures and images for: The transmission dynamics and diversity of human metapneumovirus in Peru
Source: Influenza Other Respir Viruses. 2018 Mar 30;12(4):508–13. doi: 10.1111/irv.12537 (PMC6005599; doi:10.1111/irv.12537)

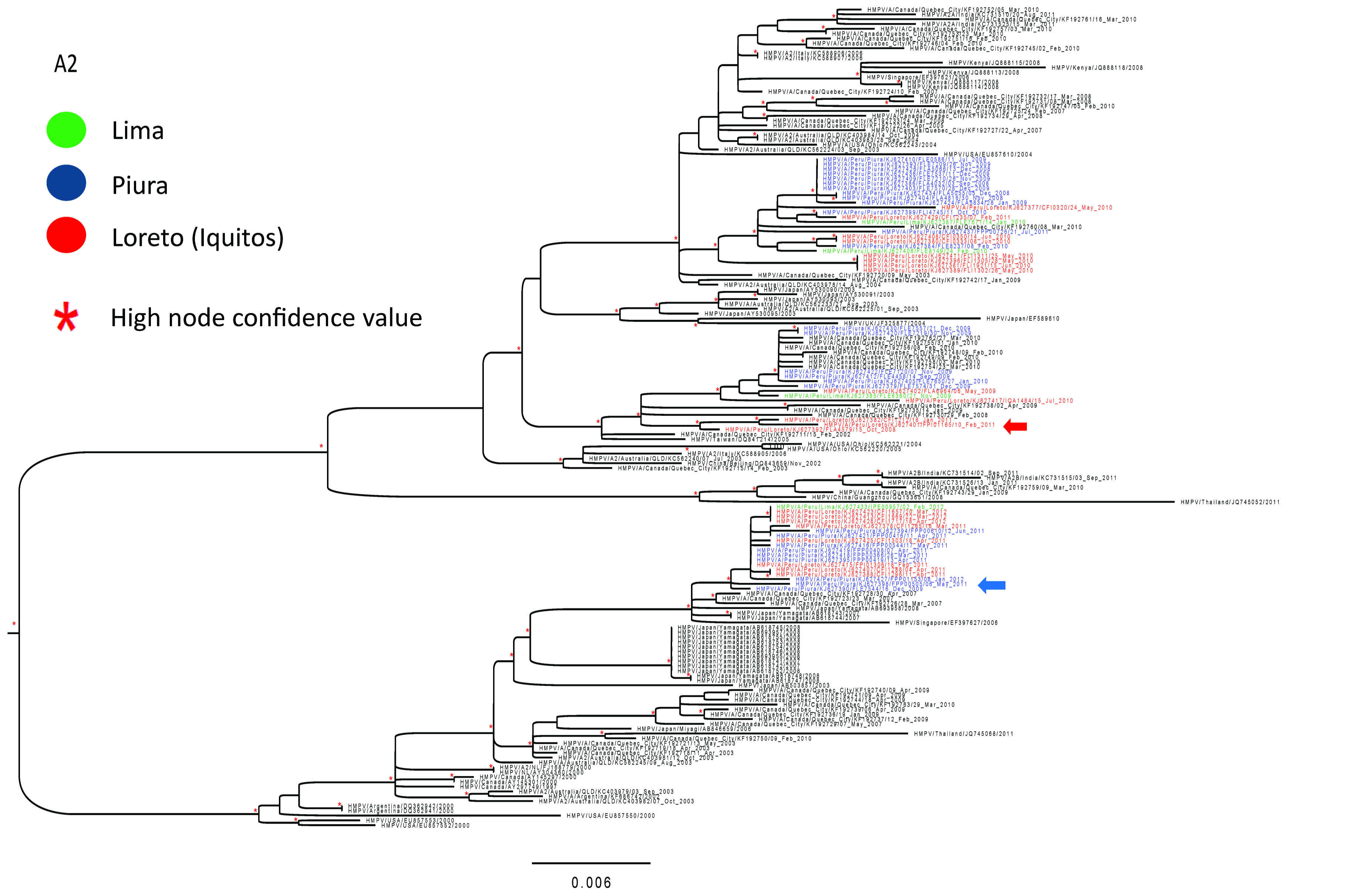

Supplement: Supplementary file 1 [file IRV-12-508-s001.tif]

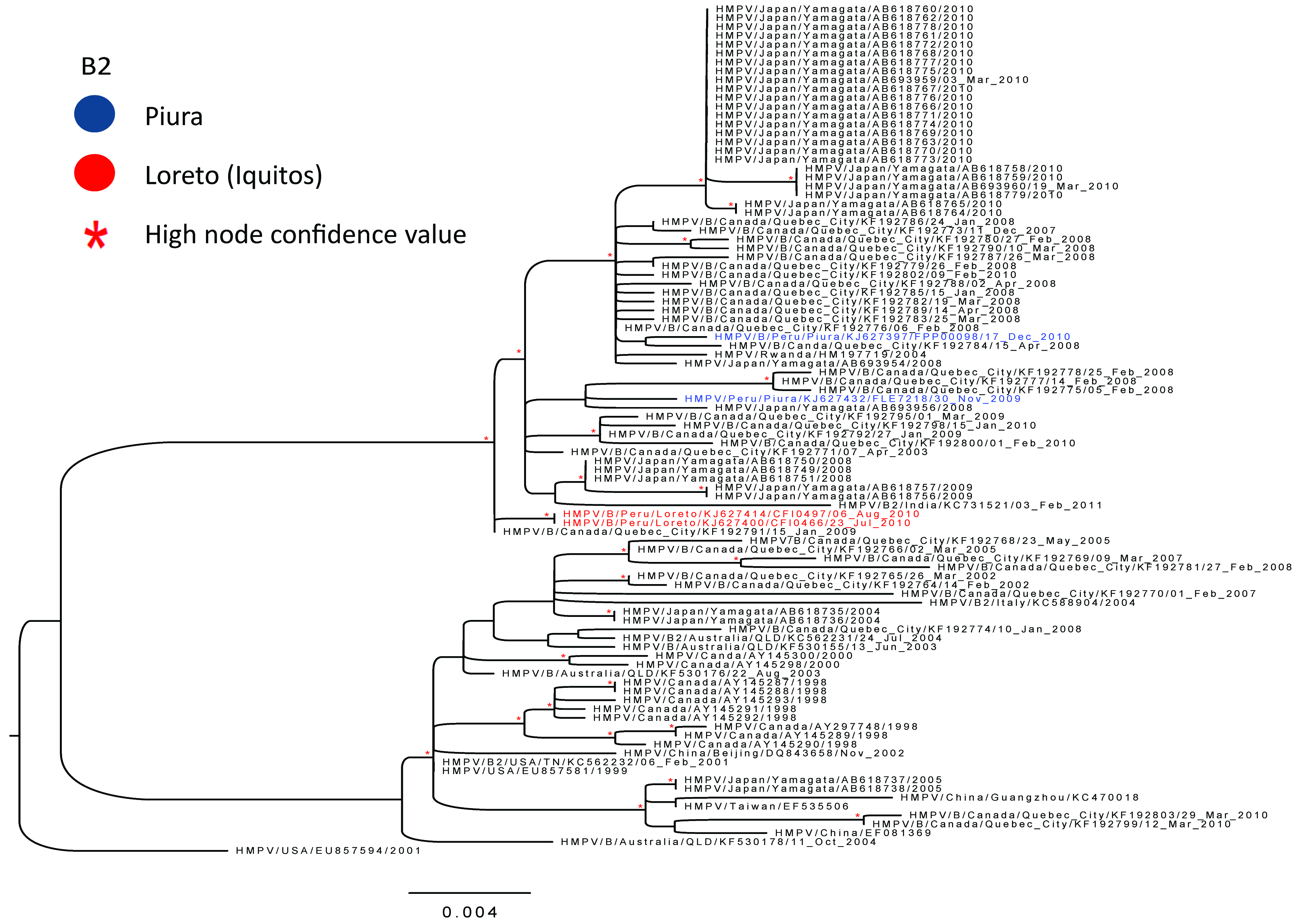

Supplement: Supplementary file 2 [file IRV-12-508-s002.tif]

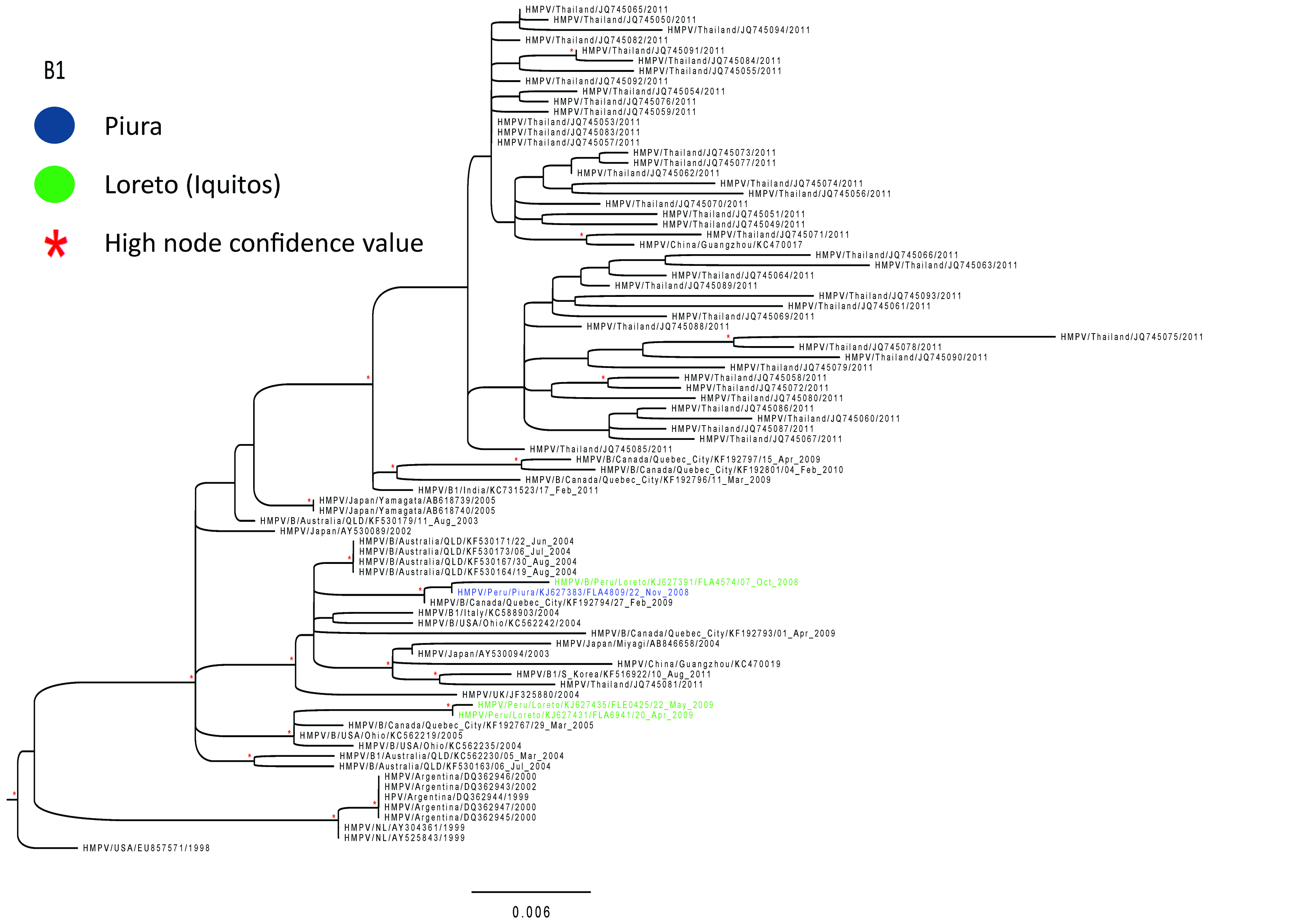

Supplement: Supplementary file 3 [file IRV-12-508-s003.tif]

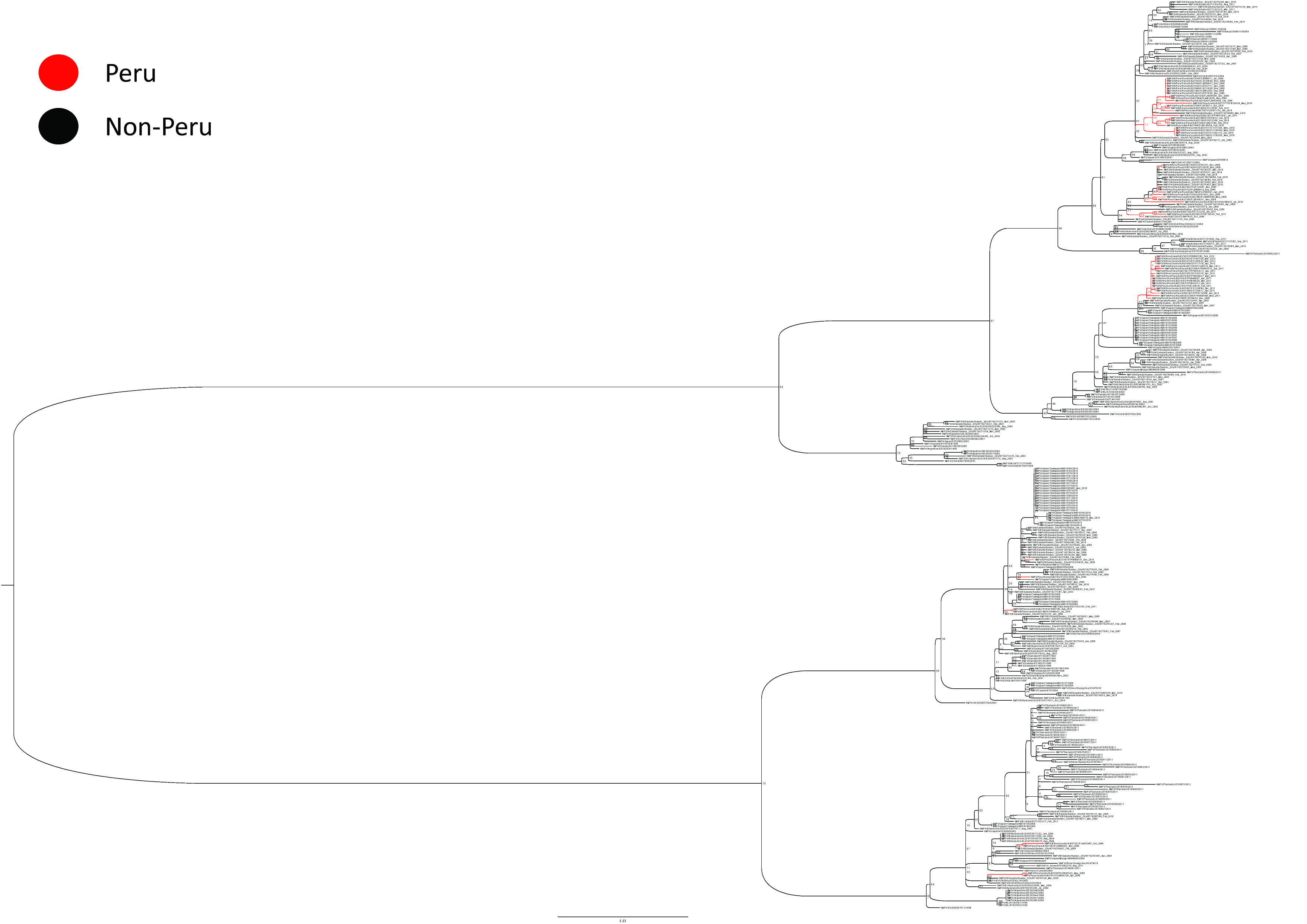

Supplement: Supplementary file 4 [file IRV-12-508-s004.tif]
