# Supplementary material for: The transmission dynamics and diversity of human metapneumovirus in Peru
Source: Influenza Other Respir Viruses. 2018 Mar 30;12(4):508–13. doi: 10.1111/irv.12537 (PMC6005599; doi:10.1111/irv.12537)
Supplement: Supplementary file 5 [file IRV-12-508-s005.docx]

**Supplementary material**

| **Table S1. Country, date and accession number of sequences used in analyses** | | | | |
| --- | --- | --- | --- | --- |
| Avian_PV/USA/AY579780 |  |  |  |  |
| HMPV/A1/Australia/QLD/KC403977/12_Aug_2003 | | |  |  |
| HMPV/A1/Australia/QLD/KC562226/05_Oct_2003 | | |  |  |
| HMPV/A1/Italy/KC588902/2001 |  |  |  |  |
| HMPV/A2/Australia/QLD/KC403978/14_Aug_2004 | | |  |  |
| HMPV/A2/Australia/QLD/KC403979/03_Sep_2003 | | |  |  |
| HMPV/A2/Australia/QLD/KC403981/12_Oct_2003 | | |  |  |
| HMPV/A2/Australia/QLD/KC403982/07_Oct_2003 | | |  |  |
| HMPV/A2/Australia/QLD/KC403983/28_Sep_2004 | | |  |  |
| HMPV/A2/Australia/QLD/KC403984/14_Oct_2004 | | |  |  |
| HMPV/A2/Australia/QLD/KC562224/03_Sep_2003 | | |  |  |
| HMPV/A2/Australia/QLD/KC562225/01_Sep_2003 | | |  |  |
| HMPV/A2/Australia/QLD/KC562240/07_Jul_2003 | | |  |  |
| HMPV/A2/Italy/KC588905/2006 |  |  |  |  |
| HMPV/A2/Italy/KC588906/2006 |  |  |  |  |
| HMPV/A2/Italy/KC588907/2006 |  |  |  |  |
| HMPV/A2/NL/FJ168779/2000 |  |  |  |  |
| HMPV/A2A/India/KC731510/20_Aug_2011 | |  |  |  |
| HMPV/A2A/India/KC731525/15_Mar_2011 | |  |  |  |
| HMPV/A2B/India/KC731514/02_Sep_2011 | |  |  |  |
| HMPV/A2B/India/KC731515/03_Sep_2011 | |  |  |  |
| HMPV/A2B/India/KC731526/13_Jan_2011 | |  |  |  |
| HMPV/A/Argentina/KF686742/2002 | |  |  |  |
| HMPV/A/Australia/QLD/KC562233/27_Aug_2003 | | |  |  |
| HMPV/A/Australia/QLD/KC562236/06_Aug_2004 | | |  |  |
| HMPV/A/Australia/QLD/KC562245/09_Aug_2003 | | |  |  |
| HMPV/A/Canada/Quebec_City/KF192711/15_Feb_2002 | | |  |  |
| HMPV/A/Canada/Quebec_City/KF192712/12_Mar_2002 | | |  |  |
| HMPV/A/Canada/Quebec_City/KF192713/24_Mar_2002 | | |  |  |
| HMPV/A/Canada/Quebec_City/KF192714/10_Feb_2003 | | |  |  |
| HMPV/A/Canada/Quebec_City/KF192715/14_Feb_2003 | | |  |  |
| HMPV/A/Canada/Quebec_City/KF192716/21_Feb_2003 | | |  |  |
| HMPV/A/Canada/Quebec_City/KF192717/31_Mar_2003 | | |  |  |
| HMPV/A/Canada/Quebec_City/KF192718/11_Apr_2003 | | |  |  |
| HMPV/A/Canada/Quebec_City/KF192719/18_Apr_2003 | | |  |  |
| HMPV/A/Canada/Quebec_City/KF192720/09_May_2003 | | |  |  |
| HMPV/A/Canada/Quebec_City/KF192721/13_May_2003 | | |  |  |
| HMPV/A/Canada/Quebec_City/KF192722/26_Apr_2005 | | |  |  |
| HMPV/A/Canada/Quebec_City/KF192723/23_Mar_2007 | | |  |  |
| HMPV/A/Canada/Quebec_City/KF192724/10_Feb_2007 | | |  |  |
| HMPV/A/Canada/Quebec_City/KF192725/24_Feb_2007 | | |  |  |
| HMPV/A/Canada/Quebec_City/KF192726/28_Mar_2007 | | |  |  |
| HMPV/A/Canada/Quebec_City/KF192727/22_Apr_2007 | | |  |  |
| HMPV/A/Canada/Quebec_City/KF192728/30_Apr_2007 | | |  |  |
| HMPV/A/Canada/Quebec_City/KF192729/07_May_2007 | | |  |  |
| HMPV/A/Canada/Quebec_City/KF192730/29_Feb_2008 | | |  |  |
| HMPV/A/Canada/Quebec_City/KF192731/08_Mar_2008 | | |  |  |
| HMPV/A/Canada/Quebec_City/KF192732/17_Mar_2008 | | |  |  |
| HMPV/A/Canada/Quebec_City/KF192733/24_Mar_2008 | | |  |  |
| HMPV/A/Canada/Quebec_City/KF192734/29_Apr_2008 | | |  |  |
| HMPV/A/Canada/Quebec_City/KF192735/14_Jan_2009 | | |  |  |
| HMPV/A/Canada/Quebec_City/KF192736/19_Jan_2009 | | |  |  |
| HMPV/A/Canada/Quebec_City/KF192737/12_Feb_2009 | | |  |  |
| HMPV/A/Canada/Quebec_City/KF192738/02_Apr_2009 | | |  |  |
| HMPV/A/Canada/Quebec_City/KF192739/06_Apr_2009 | | |  |  |
| HMPV/A/Canada/Quebec_City/KF192740/09_Apr_2009 | | |  |  |
| HMPV/A/Canada/Quebec_City/KF192741/09_Apr_2009 | | |  |  |
| HMPV/A/Canada/Quebec_City/KF192742/17_Jan_2009 | | |  |  |
| HMPV/A/Canada/Quebec_City/KF192743/29_Jan_2009 | | |  |  |
| HMPV/A/Canada/Quebec_City/KF192744/18_Apr_2009 | | |  |  |
| HMPV/A/Canada/Quebec_City/KF192745/02_Feb_2010 | | |  |  |
| HMPV/A/Canada/Quebec_City/KF192746/04_Feb_2010 | | |  |  |
| HMPV/A/Canada/Quebec_City/KF192747/05_Feb_2010 | | |  |  |
| HMPV/A/Canada/Quebec_City/KF192748/09_Feb_2010 | | |  |  |
| HMPV/A/Canada/Quebec_City/KF192749/09_Feb_2010 | | |  |  |
| HMPV/A/Canada/Quebec_City/KF192750/09_Feb_2010 | | |  |  |
| HMPV/A/Canada/Quebec_City/KF192751/16_Feb_2010 | | |  |  |
| HMPV/A/Canada/Quebec_City/KF192752/05_Mar_2010 | | |  |  |
| HMPV/A/Canada/Quebec_City/KF192753/23_Mar_2010 | | |  |  |
| HMPV/A/Canada/Quebec_City/KF192754/23_Mar_2010 | | |  |  |
| HMPV/A/Canada/Quebec_City/KF192755/31_Jan_2010 | | |  |  |
| HMPV/A/Canada/Quebec_City/KF192756/08_Feb_2010 | | |  |  |
| HMPV/A/Canada/Quebec_City/KF192757/03_Mar_2010 | | |  |  |
| HMPV/A/Canada/Quebec_City/KF192758/03_Mar_2010 | | |  |  |
| HMPV/A/Canada/Quebec_City/KF192759/09_Mar_2010 | | |  |  |
| HMPV/A/Canada/Quebec_City/KF192760/08_Mar_2010 | | |  |  |
| HMPV/A/Canada/Quebec_City/KF192761/16_Mar_2010 | | |  |  |
| HMPV/A/Canada/Quebec_City/KF192762/27_Mar_2010 | | |  |  |
| HMPV/A/Canada/Quebec_City/KF192763/29_Mar_2010 | | |  |  |
| HMPV/A/Peru/Lima/KJ627385/FLE6360/21_Nov_2009 | | |  |  |
| HMPV/A/Peru/Lima/KJ627387/FLE7671/14_Jan_2010 | | |  |  |
| HMPV/A/Peru/Lima/KJ627406/FLE8149/24_Feb_2010 | | |  |  |
| HMPV/A/Peru/Lima/KJ627433/IPE00957/02_Feb_2012 | | |  |  |
| HMPV/A/Peru/Loreto/KJ627377/CFI0320/24_May_2010 | | |  |  |
| HMPV/A/Peru/Loreto/KJ627378/CFI1265/16_Mar_2011 | | |  |  |
| HMPV/A/Peru/Loreto/KJ627380/CFI0333/08_Jun_2010 | | |  |  |
| HMPV/A/Peru/Loreto/KJ627381/FLI1921/15_Jun_2010 | | |  |  |
| HMPV/A/Peru/Loreto/KJ627382/CFI1212/18_Jan_2011 | | |  |  |
| HMPV/A/Peru/Loreto/KJ627388/CFI1296/11_Apr_2011 | | |  |  |
| HMPV/A/Peru/Loreto/KJ627389/FLI1302/26_May_2010 | | |  |  |
| HMPV/A/Peru/Loreto/KJ627392/FLA4579/15_Oct_2008 | | |  |  |
| HMPV/A/Peru/Loreto/KJ627396/FLI1305/28_May_2010 | | |  |  |
| HMPV/A/Peru/Loreto/KJ627401/FPI01165/10_Feb_2011 | | |  |  |
| HMPV/A/Peru/Loreto/KJ627402/FLA6964/06_May_2009 | | |  |  |
| HMPV/A/Peru/Loreto/KJ627407/CFI1288/04_Apr_2011 | | |  |  |
| HMPV/A/Peru/Loreto/KJ627408/CFI0350/14_Jun_2010 | | |  |  |
| HMPV/A/Peru/Loreto/KJ627411/FLI1311/25_May_2010 | | |  |  |
| HMPV/A/Peru/Loreto/KJ627413/CFI1669/22_Mar_2012 | | |  |  |
| HMPV/A/Peru/Loreto/KJ627415/FPI01306/18_Feb_2011 | | |  |  |
| HMPV/A/Peru/Loreto/KJ627417/IQA1484/15_Jul_2010 | | |  |  |
| HMPV/A/Peru/Loreto/KJ627423/CFI1657/20_Mar_2012 | | |  |  |
| HMPV/A/Peru/Loreto/KJ627425/CFI1303/18_Apr_2011 | | |  |  |
| HMPV/A/Peru/Loreto/KJ627428/CFI1717/18_Apr_2012 | | |  |  |
| HMPV/A/Peru/Loreto/KJ627429/CFI1235/07_Feb_2011 | | |  |  |
| HMPV/A/Peru/Piura/KJ627379/FLE7574/31_Dec_2009 | | |  |  |
| HMPV/A/Peru/Piura/KJ627384/FLE8237/08_Feb_2010 | | |  |  |
| HMPV/A/Peru/Piura/KJ627386/FLA4032/03_Sep_2008 | | |  |  |
| HMPV/A/Peru/Piura/KJ627390/FLE7544/16_Dec_2009 | | |  |  |
| HMPV/A/Peru/Piura/KJ627393/FLE7209/26_Nov_2009 | | |  |  |
| HMPV/A/Peru/Piura/KJ627394/FPP00610/12_Jun_2011 | | |  |  |
| HMPV/A/Peru/Piura/KJ627395/FPP00419/13_Apr_2011 | | |  |  |
| HMPV/A/Peru/Piura/KJ627398/FPP00505/06_May_2011 | | | |  |
| HMPV/A/Peru/Piura/KJ627399/FLI4745/11_Oct_2010 | | |  |  |
| HMPV/A/Peru/Piura/KJ627403/FLE7570/28_Dec_2009 | | |  |  |
| HMPV/A/Peru/Piura/KJ627405/FLE7850/27_Jan_2010 | | |  |  |
| HMPV/A/Peru/Piura/KJ627409/FLE7210/26_Nov_2009 | | |  |  |
| HMPV/A/Peru/Piura/KJ627410/FLE0586/11_Jul_2009 | | |  |  |
| HMPV/A/Peru/Piura/KJ627412/FLE4458/14_Sep_2009 | | |  |  |
| HMPV/A/Peru/Piura/KJ627416/FPP00544/17_May_2011 | | | |  |
| HMPV/A/Peru/Piura/KJ627418/FPP00366/28_Mar_2011 | | |  |  |
| HMPV/A/Peru/Piura/KJ627419/FPP00408/07_Apr_2011 | | |  |  |
| HMPV/A/Peru/Piura/KJ627420/FLE7219/30_Nov_2009 | | |  |  |
| HMPV/A/Peru/Piura/KJ627421/FPP00416/11_Apr_2011 | | |  |  |
| HMPV/A/Peru/Piura/KJ627422/FLE7120/07_Nov_2009 | | |  |  |
| HMPV/A/Peru/Piura/KJ627424/FLA5834/28_Jan_2009 | | |  |  |
| HMPV/A/Peru/Piura/KJ627426/FLA5066/13_Dec_2008 | | |  |  |
| HMPV/A/Peru/Piura/KJ627427/FPP01153/08_Jan_2012 | | |  |  |
| HMPV/A/Peru/Piura/KJ627430/FLE7557/21_Dec_2009 | | |  |  |
| HMPV/A/Peru/Piura/KJ627434/FLA5055/05_Dec_2008 | | |  |  |
| HMPV/A/Peru/Piura/KJ627436/FLE7537/11_Dec_2009 | | |  |  |
| HMPV/A/Peru/Piura/KJ627437/FPP00726/21_Jul_2011 | | |  |  |
| HMPV/A/USA/Ohio/KC562220/2005 | |  |  |  |
| HMPV/A/USA/Ohio/KC562221/2004 | |  |  |  |
| HMPV/A/USA/Ohio/KC562243/2004 | |  |  |  |
| HMPV/Argentina/DQ362937/2002 | |  |  |  |
| HMPV/Argentina/DQ362938/2002 | |  |  |  |
| HMPV/Argentina/DQ362939/1998 | |  |  |  |
| HMPV/Argentina/DQ362940/2003 | |  |  |  |
| HMPV/Argentina/DQ362941/2000 | |  |  |  |
| HMPV/Argentina/DQ362942/2000 | |  |  |  |
| HMPV/Argentina/DQ362943/2002 | |  |  |  |
| HMPV/Argentina/DQ362944/1999 | |  |  |  |
| HMPV/Argentina/DQ362945/2000 | |  |  |  |
| HMPV/Argentina/DQ362946/2000 | |  |  |  |
| HMPV/Argentina/DQ362947/2000 | |  |  |  |
| HMPV/B1/Australia/QLD/KC562230/05_Mar_2004 | | |  |  |
| HMPV/B1/India/KC731523/17_Feb_2011 | |  |  |  |
| HMPV/B1/Italy/KC588903/2004 |  |  |  |  |
| HMPV/B1/S_Korea/KF516922/10_Aug_2011 | |  |  |  |
| HMPV/B2/Australia/QLD/KC562231/24_Jul_2004 | | |  |  |
| HMPV/B2/India/KC731521/03_Feb_2011 | |  |  |  |
| HMPV/B2/Italy/KC588904/2004 |  |  |  |  |
| HMPV/B2/USA/TN/KC562232/06_Feb_2001 | |  |  |  |
| HMPV/B/Australia/QLD/KF530155/13_Jun_2003 | | |  |  |
| HMPV/B/Australia/QLD/KF530163/06_Jul_2004 | | |  |  |
| HMPV/B/Australia/QLD/KF530164/19_Aug_2004 | | |  |  |
| HMPV/B/Australia/QLD/KF530167/30_Aug_2004 | | |  |  |
| HMPV/B/Australia/QLD/KF530171/22_Jun_2004 | | |  |  |
| HMPV/B/Australia/QLD/KF530173/06_Jul_2004 | | |  |  |
| HMPV/B/Australia/QLD/KF530176/22_Aug_2003 | | |  |  |
| HMPV/B/Australia/QLD/KF530178/11_Oct_2004 | | |  |  |
| HMPV/B/Australia/QLD/KF530179/11_Aug_2003 | | |  |  |
| HMPV/B/Canada/Quebec_City/KF192764/14_Feb_2002 | | |  |  |
| HMPV/B/Canada/Quebec_City/KF192765/26_Mar_2002 | | |  |  |
| HMPV/B/Canada/Quebec_City/KF192766/02_Mar_2005 | | |  |  |
| HMPV/B/Canada/Quebec_City/KF192767/29_Mar_2005 | | |  |  |
| HMPV/B/Canada/Quebec_City/KF192768/23_May_2005 | | |  |  |
| HMPV/B/Canada/Quebec_City/KF192769/09_Mar_2007 | | |  |  |
| HMPV/B/Canada/Quebec_City/KF192770/01_Feb_2007 | | |  |  |
| HMPV/B/Canada/Quebec_City/KF192771/07_Apr_2003 | | |  |  |
| HMPV/B/Canada/Quebec_City/KF192773/11_Dec_2007 | | |  |  |
| HMPV/B/Canada/Quebec_City/KF192774/10_Jan_2008 | | |  |  |
| HMPV/B/Canada/Quebec_City/KF192775/05_Feb_2008 | | |  |  |
| HMPV/B/Canada/Quebec_City/KF192776/06_Feb_2008 | | |  |  |
| HMPV/B/Canada/Quebec_City/KF192777/14_Feb_2008 | | |  |  |
| HMPV/B/Canada/Quebec_City/KF192778/25_Feb_2008 | | |  |  |
| HMPV/B/Canada/Quebec_City/KF192779/26_Feb_2008 | | |  |  |
| HMPV/B/Canada/Quebec_City/KF192780/27_Feb_2008 | | |  |  |
| HMPV/B/Canada/Quebec_City/KF192781/27_Feb_2008 | | |  |  |
| HMPV/B/Canada/Quebec_City/KF192782/19_Mar_2008 | | |  |  |
| HMPV/B/Canada/Quebec_City/KF192783/25_Mar_2008 | | |  |  |
| HMPV/B/Canada/Quebec_City/KF192785/15_Jan_2008 | | |  |  |
| HMPV/B/Canada/Quebec_City/KF192786/24_Jan_2008 | | |  |  |
| HMPV/B/Canada/Quebec_City/KF192787/26_Mar_2008 | | |  |  |
| HMPV/B/Canada/Quebec_City/KF192788/02_Apr_2008 | | |  |  |
| HMPV/B/Canada/Quebec_City/KF192789/14_Apr_2008 | | |  |  |
| HMPV/B/Canada/Quebec_City/KF192790/10_Mar_2008 | | |  |  |
| HMPV/B/Canada/Quebec_City/KF192791/15_Jan_2009 | | |  |  |
| HMPV/B/Canada/Quebec_City/KF192792/27_Jan_2009 | | |  |  |
| HMPV/B/Canada/Quebec_City/KF192793/01_Apr_2009 | | |  |  |
| HMPV/B/Canada/Quebec_City/KF192794/27_Feb_2009 | | |  |  |
| HMPV/B/Canada/Quebec_City/KF192795/01_Mar_2009 | | |  |  |
| HMPV/B/Canada/Quebec_City/KF192796/11_Mar_2009 | | |  |  |
| HMPV/B/Canada/Quebec_City/KF192797/15_Apr_2009 | | |  |  |
| HMPV/B/Canada/Quebec_City/KF192798/15_Jan_2010 | | |  |  |
| HMPV/B/Canada/Quebec_City/KF192799/12_Mar_2010 | | |  |  |
| HMPV/B/Canada/Quebec_City/KF192800/01_Feb_2010 | | |  |  |
| HMPV/B/Canada/Quebec_City/KF192801/04_Feb_2010 | | |  |  |
| HMPV/B/Canada/Quebec_City/KF192802/09_Feb_2010 | | |  |  |
| HMPV/B/Canada/Quebec_City/KF192803/29_Mar_2010 | | |  |  |
| HMPV/B/Canda/Quebec_City/KF192784/15_Apr_2008 | | |  |  |
| HMPV/B/Peru/Loreto/KJ627391/FLA4574/07_Oct_2008 | | |  |  |
| HMPV/B/Peru/Loreto/KJ627400/CFI0466/23_Jul_2010 | | |  |  |
| HMPV/B/Peru/Loreto/KJ627414/CFI0497/06_Aug_2010 | | |  |  |
| HMPV/B/Peru/Piura/KJ627397/FPP00098/17_Dec_2010 | | |  |  |
| HMPV/B/USA/Ohio/KC562219/2005 | |  |  |  |
| HMPV/B/USA/Ohio/KC562235/2004 | |  |  |  |
| HMPV/B/USA/Ohio/KC562242/2004 | |  |  |  |
| HMPV/Canada/AY145287/1998 |  |  |  |  |
| HMPV/Canada/AY145288/1998 |  |  |  |  |
| HMPV/Canada/AY145289/1998 |  |  |  |  |
| HMPV/Canada/AY145290/1998 |  |  |  |  |
| HMPV/Canada/AY145291/1998 |  |  |  |  |
| HMPV/Canada/AY145292/1998 |  |  |  |  |
| HMPV/Canada/AY145293/1998 |  |  |  |  |
| HMPV/Canada/AY145294/1999 |  |  |  |  |
| HMPV/Canada/AY145297/2000 |  |  |  |  |
| HMPV/Canada/AY145298/2000 |  |  |  |  |
| HMPV/Canada/AY145299/2000 |  |  |  |  |
| HMPV/Canada/AY145301/2000 |  |  |  |  |
| HMPV/Canada/AY297748/1998 |  |  |  |  |
| HMPV/Canada/AY297749/1997 |  |  |  |  |
| HMPV/Canda/AY145300/2000 |  |  |  |  |
| HMPV/China/Beijing/DQ843658/Nov_2002 | |  |  |  |
| HMPV/China/Beijing/DQ843659/Nov_2002 | |  |  |  |
| HMPV/China/EF081369 |  |  |  |  |
| HMPV/China/Guangzhou/GQ153651/2008 | |  |  |  |
| HMPV/China/Guangzhou/KC470017 | |  |  |  |
| HMPV/China/Guangzhou/KC470018 | |  |  |  |
| HMPV/China/Guangzhou/KC470019 | |  |  |  |
| HMPV/Japan/AB503857/2003 |  |  |  |  |
| HMPV/Japan/AY530089/2002 |  |  |  |  |
| HMPV/Japan/AY530090/2003 |  |  |  |  |
| HMPV/Japan/AY530091/2003 |  |  |  |  |
| HMPV/Japan/AY530092/2003 |  |  |  |  |
| HMPV/Japan/AY530093/2003 |  |  |  |  |
| HMPV/Japan/AY530094/2003 |  |  |  |  |
| HMPV/Japan/AY530095/2003 |  |  |  |  |
| HMPV/Japan/EF589610 |  |  |  |  |
| HMPV/Japan/Miyagi/AB846658/2004 | |  |  |  |
| HMPV/Japan/Miyagi/AB846659/2006 | |  |  |  |
| HMPV/Japan/Yamagata/AB618735/2004 | |  |  |  |
| HMPV/Japan/Yamagata/AB618736/2004 | |  |  |  |
| HMPV/Japan/Yamagata/AB618737/2005 | |  |  |  |
| HMPV/Japan/Yamagata/AB618738/2005 | |  |  |  |
| HMPV/Japan/Yamagata/AB618739/2005 | |  |  |  |
| HMPV/Japan/Yamagata/AB618740/2005 | |  |  |  |
| HMPV/Japan/Yamagata/AB618741/2007 | |  |  |  |
| HMPV/Japan/Yamagata/AB618742/2007 | |  |  |  |
| HMPV/Japan/Yamagata/AB618743/2007 | |  |  |  |
| HMPV/Japan/Yamagata/AB618744/2007 | |  |  |  |
| HMPV/Japan/Yamagata/AB618745/2008 | |  |  |  |
| HMPV/Japan/Yamagata/AB618746/2008 | |  |  |  |
| HMPV/Japan/Yamagata/AB618747/2008 | |  |  |  |
| HMPV/Japan/Yamagata/AB618748/2008 | |  |  |  |
| HMPV/Japan/Yamagata/AB618749/2008 | |  |  |  |
| HMPV/Japan/Yamagata/AB618750/2008 | |  |  |  |
| HMPV/Japan/Yamagata/AB618751/2008 | |  |  |  |
| HMPV/Japan/Yamagata/AB618752/2008 | |  |  |  |
| HMPV/Japan/Yamagata/AB618753/2008 | |  |  |  |
| HMPV/Japan/Yamagata/AB618754/2008 | |  |  |  |
| HMPV/Japan/Yamagata/AB618755/2008 | |  |  |  |
| HMPV/Japan/Yamagata/AB618756/2009 | |  |  |  |
| HMPV/Japan/Yamagata/AB618757/2009 | |  |  |  |
| HMPV/Japan/Yamagata/AB618758/2010 | |  |  |  |
| HMPV/Japan/Yamagata/AB618759/2010 | |  |  |  |
| HMPV/Japan/Yamagata/AB618760/2010 | |  |  |  |
| HMPV/Japan/Yamagata/AB618761/2010 | |  |  |  |
| HMPV/Japan/Yamagata/AB618762/2010 | |  |  |  |
| HMPV/Japan/Yamagata/AB618763/2010 | |  |  |  |
| HMPV/Japan/Yamagata/AB618764/2010 | |  |  |  |
| HMPV/Japan/Yamagata/AB618765/2010 | |  |  |  |
| HMPV/Japan/Yamagata/AB618766/2010 | |  |  |  |
| HMPV/Japan/Yamagata/AB618767/2010 | |  |  |  |
| HMPV/Japan/Yamagata/AB618768/2010 | |  |  |  |
| HMPV/Japan/Yamagata/AB618769/2010 | |  |  |  |
| HMPV/Japan/Yamagata/AB618770/2010 | |  |  |  |
| HMPV/Japan/Yamagata/AB618771/2010 | |  |  |  |
| HMPV/Japan/Yamagata/AB618772/2010 | |  |  |  |
| HMPV/Japan/Yamagata/AB618773/2010 | |  |  |  |
| HMPV/Japan/Yamagata/AB618774/2010 | |  |  |  |
| HMPV/Japan/Yamagata/AB618775/2010 | |  |  |  |
| HMPV/Japan/Yamagata/AB618776/2010 | |  |  |  |
| HMPV/Japan/Yamagata/AB618777/2010 | |  |  |  |
| HMPV/Japan/Yamagata/AB618778/2010 | |  |  |  |
| HMPV/Japan/Yamagata/AB618779/2010 | |  |  |  |
| HMPV/Japan/Yamagata/AB693954/2008 | |  |  |  |
| HMPV/Japan/Yamagata/AB693955/2008 | |  |  |  |
| HMPV/Japan/Yamagata/AB693956/2008 | |  |  |  |
| HMPV/Japan/Yamagata/AB693957/2008 | |  |  |  |
| HMPV/Japan/Yamagata/AB693958/2008 | |  |  |  |
| HMPV/Japan/Yamagata/AB693959/03_Mar_2010 | | |  |  |
| HMPV/Japan/Yamagata/AB693960/19_Mar_2010 | | |  |  |
| HMPV/Kenya/JQ888113/2008 |  |  |  |  |
| HMPV/Kenya/JQ888114/2008 |  |  |  |  |
| HMPV/Kenya/JQ888115/2008 |  |  |  |  |
| HMPV/Kenya/JQ888117/2008 |  |  |  |  |
| HMPV/Kenya/JQ888118/2008 |  |  |  |  |
| HMPV/NL/AF371337/2000 |  |  |  |  |
| HMPV/NL/AY304360/2000 |  |  |  |  |
| HMPV/NL/AY304361/1999 |  |  |  |  |
| HMPV/NL/AY525843/1999 |  |  |  |  |
| HMPV/Peru/Loreto/KJ627431/FLA6941/20_Apr_2009 | | |  |  |
| HMPV/Peru/Loreto/KJ627435/FLE0425/22_May_2009 | | |  |  |
| HMPV/Peru/Piura/KJ627383/FLA4809/22_Nov_2008 | | |  |  |
| HMPV/Peru/Piura/KJ627404/FLA4816/30_Nov_2008 | | |  |  |
| HMPV/Peru/Piura/KJ627432/FLE7218/30_Nov_2009 | | |  |  |
| HMPV/Rwanda/HM197719/2004 |  |  |  |  |
| HMPV/Singapore/EF397621/2006 |  |  |  |  |
| HMPV/Singapore/EF397627/2006 |  |  |  |  |
| HMPV/Taiwan/DQ841214/2005 |  |  |  |  |
| HMPV/Taiwan/EF535506 |  |  |  |  |
| HMPV/Thailand/JQ745049/2011 |  |  |  |  |
| HMPV/Thailand/JQ745050/2011 |  |  |  |  |
| HMPV/Thailand/JQ745051/2011 |  |  |  |  |
| HMPV/Thailand/JQ745052/2011 |  |  |  |  |
| HMPV/Thailand/JQ745053/2011 |  |  |  |  |
| HMPV/Thailand/JQ745054/2011 |  |  |  |  |
| HMPV/Thailand/JQ745055/2011 |  |  |  |  |
| HMPV/Thailand/JQ745056/2011 |  |  |  |  |
| HMPV/Thailand/JQ745057/2011 |  |  |  |  |
| HMPV/Thailand/JQ745058/2011 |  |  |  |  |
| HMPV/Thailand/JQ745059/2011 |  |  |  |  |
| HMPV/Thailand/JQ745060/2011 |  |  |  |  |
| HMPV/Thailand/JQ745061/2011 |  |  |  |  |
| HMPV/Thailand/JQ745062/2011 |  |  |  |  |
| HMPV/Thailand/JQ745063/2011 |  |  |  |  |
| HMPV/Thailand/JQ745064/2011 |  |  |  |  |
| HMPV/Thailand/JQ745065/2011 |  |  |  |  |
| HMPV/Thailand/JQ745066/2011 |  |  |  |  |
| HMPV/Thailand/JQ745067/2011 |  |  |  |  |
| HMPV/Thailand/JQ745068/2011 |  |  |  |  |
| HMPV/Thailand/JQ745069/2011 |  |  |  |  |
| HMPV/Thailand/JQ745070/2011 |  |  |  |  |
| HMPV/Thailand/JQ745071/2011 |  |  |  |  |
| HMPV/Thailand/JQ745072/2011 |  |  |  |  |
| HMPV/Thailand/JQ745073/2011 |  |  |  |  |
| HMPV/Thailand/JQ745074/2011 |  |  |  |  |
| HMPV/Thailand/JQ745075/2011 |  |  |  |  |
| HMPV/Thailand/JQ745076/2011 |  |  |  |  |
| HMPV/Thailand/JQ745077/2011 |  |  |  |  |
| HMPV/Thailand/JQ745078/2011 |  |  |  |  |
| HMPV/Thailand/JQ745079/2011 |  |  |  |  |
| HMPV/Thailand/JQ745080/2011 |  |  |  |  |
| HMPV/Thailand/JQ745081/2011 |  |  |  |  |
| HMPV/Thailand/JQ745082/2011 |  |  |  |  |
| HMPV/Thailand/JQ745083/2011 |  |  |  |  |
| HMPV/Thailand/JQ745084/2011 |  |  |  |  |
| HMPV/Thailand/JQ745085/2011 |  |  |  |  |
| HMPV/Thailand/JQ745086/2011 |  |  |  |  |
| HMPV/Thailand/JQ745087/2011 |  |  |  |  |
| HMPV/Thailand/JQ745088/2011 |  |  |  |  |
| HMPV/Thailand/JQ745089/2011 |  |  |  |  |
| HMPV/Thailand/JQ745090/2011 |  |  |  |  |
| HMPV/Thailand/JQ745091/2011 |  |  |  |  |
| HMPV/Thailand/JQ745092/2011 |  |  |  |  |
| HMPV/Thailand/JQ745093/2011 |  |  |  |  |
| HMPV/Thailand/JQ745094/2011 |  |  |  |  |
| HMPV/UK/JF325877/2004 |  |  |  |  |
| HMPV/UK/JF325880/2004 |  |  |  |  |
| HMPV/USA/EU857545/1999 |  |  |  |  |
| HMPV/USA/EU857550/2000 |  |  |  |  |
| HMPV/USA/EU857552/2000 |  |  |  |  |
| HMPV/USA/EU857553/2000 |  |  |  |  |
| HMPV/USA/EU857571/1998 |  |  |  |  |
| HMPV/USA/EU857581/1999 |  |  |  |  |
| HMPV/USA/EU857594/2001 |  |  |  |  |
| HMPV/USA/EU857609/2003 |  |  |  |  |
| HMPV/USA/EU857610/2004 |  |  |  |  |

| **Table S2. Bayesian tip-association significance testing for clustering of Peruvian HMPV strains by geographic location (inferred by strict clock model)** | | | | | | | | | |
| --- | --- | --- | --- | --- | --- | --- | --- | --- | --- |
|  | AI observed | AI expected^a^ | p- value^b^ | PS | PS, | p-value^b^ | Mean MC | Mean MC | p-value^b^ |
|  | (95% CI)^a^ |  |  | observed | expected^a^ |  | (95% CI), observed | (95% CI), expected |  |
|  |  |  |  | (95% CI)^a^ |  |  |  |  |  |
| All locations | 1.99 (1.54 - 2.43) | 3.86 (3.00 - 4.70) | <0.001 | 16.76 (15.0 - 18.0) | 22.78 (19.98 - 25.45) | <0.001 | _ | _ | _ |
| Lima^c^ | _ | _ | _ | _ | _ | _ | 1.00 (1.0 - 1.0) | 1.12 (1.0 - 2.00) | >0.99 |
| Iquitos | _ | _ | _ | _ | _ | _ | 4.00 (4.0 - 4.0) | 2.90 (2.01 -4.07) | 0.13 |
| Piura | _ | _ | _ | _ | _ | _ | 10.00 (10.0 - 10.0) | 3.43 (2.36 -5.14) | <0.01 |
| ^a^Association Index (AI) and Parsimony Scores (PS) for 'All locations'. Maximum Clade size (MC) for all locations. | | | | | | | | | |
| bp-value is the proportion of trees from the null distribution equal to, or more extreme than, the median posterior of the statistic  cAnalysis based on 4 sequences only  **Table S3. Primers used in whole genome sequencing of human metapneumovirus**   \| Genome coverage (kbp) \| Forward Primer Name \| Forward Primer Sequence \| TM \| Reverse Primer Name \| Reverse Primer Sequence \| TM \| \| --- \| --- \| --- \| --- \| --- \| --- \| --- \| \| 1-4.1 \| MPV_7F_v3 \| GCGAAAAAAACGCGTATAAATTARRTT \| 59-65 \| MPV_4154R_v3 \| CT GTG CTR ACT TTG CAY GG \| 59-65 \| \| 4-8.1 \| MPV_4082F_v3 \| ACAGCAGCRGGRATYAATGT \| 60 \| MPV_8158R_v3 \| TA GTA CTG AAY TGA GCA TGY TCA G \| 60 \| \| 7.8-11.8 \| MPV_7897F_v3 \| AACTGTTAACATGGAAAGATGTGATG \| 61 \| MPV_11125R_v3 \| TAA GCT GGA ACW GAW GCT G \| 60 \| \| 9.8-13.5 \| MPV_9804F_v3 \| TCAATAGGGAGTCTRTGTCARGAA \| 60-65 \| MPV_13488R_v3 \| G GCA AAA AAA CCG TAT ACA TCC \| 60 \| | | | | | | | | | |

**Figure S1.** High resolution trees of clade A2 taken from Figure 1 for clarity. Scale bar indicates number of nucleotide substitutions per site, and the red asterisk indicates a node bootstrap value ≥ 70. Blue and red arrows indicate putative periods of multi-year viral persistence in Piura and Loreto (Iquitos) respectively. Sequences from Lima, Piura and Loreto (Iquitos) are colored green, blue and red respectively.

**Figure S2.** High resolution trees of clade B2 taken from Figure 1 for clarity. Scale bar indicates number of nucleotide substitutions per site, and the red asterisk indicates a node bootstrap value ≥ 70. Sequences from Piura and Loreto (Iquitos) are colored blue and red respectively.

**Figure S3.** High resolution trees of clade B1 taken from Figure 1 for clarity. Scale bar indicates number of nucleotide substitutions per site, and the red asterisk indicates a node bootstrap value ≥ 70. Sequences from Piura and Loreto (Iquitos) are colored blue and green respectively.

**Figure S4.** Maximum likelihood inferred phylogeny of Peru and reference global HMPV F-gene sequences, rooted by avian pneumovirus group-C outgroup (GenBank accession AY579780, removed for clarity). Scale bar indicates number of nucleotide substitutions per site and clade identities are indicated. All tips are labelled and bootstrap values shown.
